# 3D Geometrical Inspection of Complex Geometry Parts Using a Novel Laser Triangulation Sensor and a Robot

**DOI:** 10.3390/s110100090

**Published:** 2010-12-23

**Authors:** Francisco Javier Brosed, Juan José Aguilar, David Guillomía, Jorge Santolaria

**Affiliations:** Design and Manufacturing Engineering Department, University of Zaragoza. María de Luna, 3; E-50018, Zaragoza, Spain; E-Mails: fjbrosed@unizar.es (F.J.B); daguisan@unizar.es (D.G.); jsmazo@unizar.es (J.S.)

**Keywords:** optical measurement for geometrical quantity evaluation, triangulation laser probe, in-process measurement, robot, self-recalibration

## Abstract

This article discusses different non contact 3D measuring strategies and presents a model for measuring complex geometry parts, manipulated through a robot arm, using a novel vision system consisting of a laser triangulation sensor and a motorized linear stage. First, the geometric model incorporating an automatic simple module for long term stability improvement will be outlined in the article. The new method used in the automatic module allows the sensor set up, including the motorized linear stage, for the scanning avoiding external measurement devices. In the measurement model the robot is just a positioning of parts with high repeatability. Its position and orientation data are not used for the measurement and therefore it is not directly “coupled” as an active component in the model. The function of the robot is to present the various surfaces of the workpiece along the measurement range of the vision system, which is responsible for the measurement. Thus, the whole system is not affected by the robot own errors following a trajectory, except those due to the lack of static repeatability. For the indirect link between the vision system and the robot, the original model developed needs only one first piece measuring as a “zero” or master piece, known by its accurate measurement using, for example, a Coordinate Measurement Machine. The strategy proposed presents a different approach to traditional laser triangulation systems on board the robot in order to improve the measurement accuracy, and several important cues for self-recalibration are explored using only a master piece. Experimental results are also presented to demonstrate the technique and the final 3D measurement accuracy.

## 1. Introduction

In order to reduce time and costs while maintaining a good accuracy level there is a growing trend towards the use of measurement systems based on industrial vision for flexible automated 100% inspection of parts in sectors such as the automotive industry [1–5].

Within the group of industrial vision-based sensors, Light-Structured-Based systems (LSBs) are widespread for product geometrical inspection because of their accuracy and flexibility. LSBs systems are able to obtain 3D coordinates from a laser line projection on the measurement surface with high data acquisition speed and has been applied on the automotive, aeronautics and molds sectors, and applications related to heritage conservation and general measurements of industrial components [1–12]. Many types of LSB systems are available today, however the design of LSBs systems needs to take into consideration many factors such as accuracy, speed, working volume, reliability, and cost [1]. These factors often need to be carefully balanced for any particular application. There currently exists no industrial vision system capable of handling all tasks in every application domain. Only after the requirements in a particular application are specified, can the appropriate decisions for the design and development of such a system be taken. Nevertheless, the positioning and orientation ability between the scanning system and the measurement surface limit the scanning range. Different applications with this kind of devices mounted in measurement instruments as Coordinate Measurement Machines (CMMs) or Articulated Arm Coordinate Measurement Machines have been developed to solve range problems [13–19]. In particular, Laser Triangulation Sensors (LTSs) are nowadays the most commonly used non-contact sensors in traditional dimensional metrology and quality control tasks equipment. By combining an industrial robot and a LTS, flexibility and speed are provided to the measurement process [2,20–24] including in some cases external rotary axis [20].

Before using a robot and a LTS for measurement, usually two kind of calibration have to be performed. Firstly, the LTS calibration (intrinsic calibration) obtaining the relationship between the global frame of the camera (3D) and the frame of the projected image in the camera sensor (2D) [25]. The geometrical characteristics of the laser beam (a plane in this case) are obtained in the intrinsic calibration too.

Secondly, obtaining the relative position between the global frame of the LTS, defined in the intrinsic calibration, and the global frame of the robot it is needed (extrinsic calibration). The LTS could be mounted in the end effector of the robot as a tool and the TCP (Tool Centre Point) calibration could be considered as a robot hand-eye calibration. Several authors propose solutions for the robot hand-eye calibration (equivalent to the extrinsic calibration when the LTS is mounted in the end effector of the robot) using linear [26–29] and non-linear solutions [30,31]. Other authors propose to grasp the part being verified with the robot and fix the LTS in the base frame [23].

This paper presents a high accuracy non-contact measurement system involving a novel sensor (LTS) mounted on a Motorized Linear Stage (MLS) for digitalize surfaces and a robot manipulator to positioning different surfaces of the part in the field of view of the LTS allowing the scanning process. The model and calibration process of the system are described, as well as the proposed method for calculate and validate the movement direction of the MLS, which is needed for the surfaces reconstruction. Finally, the measurement model for the reconstruction of different surfaces in the global frame of the part is presented with the results of the test performed with a complex geometry part, in order to validating the measurement model.

The calculation method for the movement direction of the MLS avoids external measurement devices, like a CMM or a Laser Tracker, measuring the position of the MLS in the global frame of the LTS unlike several methods proposed in the literature [13,32]. The method allows the LTS self-recalibration, using a gauge object, and enables the calibration performance on the inspection line.

Another novelty presented in this paper is the measurement model developed for reconstructing different surfaces of the part in the global frame of the LTS without the robot positioning data, and so on, without the robot positioning inaccuracy. The presented model measures a master piece, as system initialization, to calculate de variation of the robot position for all the surfaces, taking advantage of the robot good positioning repeatability (a repeatability test is performed to verify the positioning repeatability) and avoiding the robot lack of accuracy and the extrinsic calibration performance.

## 2. Sensor Design

The specifications required for the application and a discussion about the need of the self-design of the LTS is pointed out firstly in this section. After that, the design of the LTS is analyzed and the devices that allow the scan of different part surfaces are shown.

### 2.1. Specifications

The design starting point of the LTS should be the definition of the measurement specifications. The characteristics of the elements to be measured and its tolerances are shown in Table 1.

The state of the art of the LSB systems has been widely reviewed in the literature [1–12]. There are a high number of laser triangulation probes available, but most of those are general purpose and their specifications do not fit with the ones required for the 100% flexible and automated 3D geometrical inspection of complex geometry parts with the characteristics and tolerances shown in Table 1. A high precision sensor is needed but, the data acquisition velocity also has to be enough to allow the inspection of the 100% of the production. Although there are some sensors with the adequate precision, the data acquisition velocity of these sensors is not enough for the application.

In order to obtain the adequate system, a specific LTS design is needed to ensure the correct inspection of parts combining relatively wide surfaces and small holes, all subject to tight tolerances. The selected components must meet some special features to suit with the specifications. For example, the laser illumination should generate a plane (a line in mage) instead of a line (a point in image) to increase the data acquisition speed; and the spatial position of the hardware should be defined to improve the resolution of the sensor in the measurement of flatness and the position of the holes.

### 2.2. Components

The LTS is composed by two cameras, with a high resolution lens and an interferential filter each one, and a laser diode with a non-Gaussian laser line generator. Hardware characteristics are shown in Table 2. The LTS is mounted in a MLS allowing the digitalization of surfaces along the MLS travel range (250 mm).

### 2.3. Geometry

**Figure 1.**
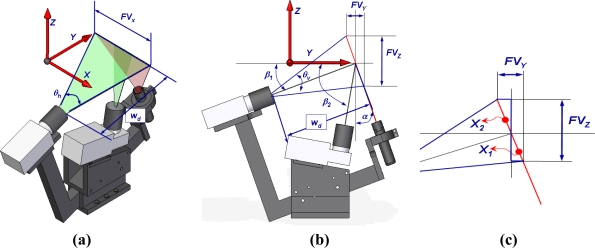
Influence of the geometrical parameters in the field of view of the LTS. **(a)** Field of view in *X* axis. **(b)** Field of view in *Y* and *Z* axis **(c)** Two components detailed decomposition of the field of view in *Y* and *Z* directions.

The spatial position and orientation of the optical elements affect the field of view of the system (Figure 1) and consequently, fixed the camera characteristics, affect the resolution too. The influence of the geometry of the LTS in these measurement characteristics has been studied to determine the best spatial configuration of the hardware, in order to manufacture a high precision stand to allocate the camera and the laser generator.

The field of view in *X* axis defines the maximum width of the measurement and is calculated from the values *w_d_* (working distance of the camera) and *θ_h_* (horizontal angle of the lens) (Equation 1):

(1) $${\mathrm{FV}}_{X}=2\cdot w_{d}\cdot \mathrm{tg}\cdot \left(\frac{\theta_{h}}{2}\right)$$

The field of view of the camera in *Y* and *Z* direction [shown in Figure 1(b)] can be calculated as the sum of *X_1_* and *X_2_* components in *Y* and *Z* axis [Figure 1(c)]. *X_1_* and *X_2_* can also be related with the geometrical parameters of the LTS as shown in Equations (2) and (3):

(2) $$\frac{w_{d}}{\text{sin}\left(180-\left(90-\alpha +\beta \right)-\frac{\theta_{v}}{2}\right)}=\frac{X_{1}}{\text{sin}\left(\frac{\theta_{v}}{2}\right)}$$

(3) $$\frac{w_{d}}{\text{sin}\left(180-\left(90-\beta +\alpha \right)-\frac{\theta_{v}}{2}\right)}=\frac{X_{2}}{\text{sin}\left(\frac{\theta_{v}}{2}\right)}$$

**Figure 2.**
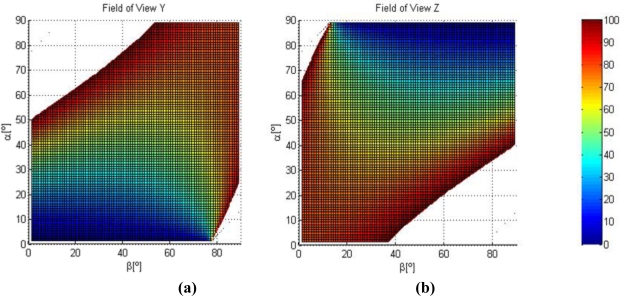
**(a)** Field of view Y coordinate [mm]. **(b)** Field of view Z coordinate [mm].

The influence of *α* (angle between laser and the vertical) and *β* (angle between camera and the horizontal) in the field of view in *Y* and *Z* direction is analysed, once the working distance, *w_d_*, is fixed from the initial specifications of the field of view in *X* direction and the lens characteristics (*θ_h_*), Figure 2.

In order to obtain adequate resolution values in *Y* (to measure element position), and in *Z* (to measure surface flatness) directions, low field of view values are searched.

**Figure 3.**
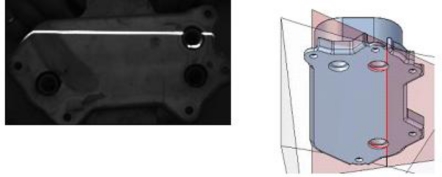
Laser reflections in the wall of the hole, *α* = 70°.

A test with different *α* values has been performed. In this test is shown that high *α* values result in laser reflections in the wall of the hole and this effect generates localization inaccuracy (Figure 3). *α* = 20° is defined to avoid laser reflections and *β* = *α* is assigned for minimize the field of view in *Y* and *Z*.

**Figure 4.**
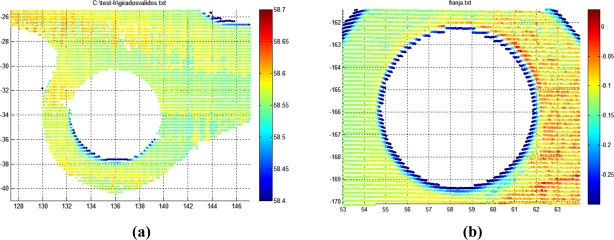
Scanned cloud points of a countersunk hole. The colour of the points indicate the distance to the surface in millimetres. **(a)** *α* = 20° & *β* = 20°. **(b)** *α* = 20° & *β* = 80°.

The assigned *α* ensures the laser light illumination in the limit edge between the wall of the hole and the bevel zone of the countersunk hole. This contour defines the hole position and diameter. In this conditions, a second camera (*β* = 80°) is needed to capture the laser incidence on the contour as is shown in the reconstructed cloud points of the scanning test using different *β* values, Figure 4.

The image resolution obtained with the devices final positions (*α* = 20°, *β_1_* = 10° & *β_2_* = 80°) are shown in Table 3 according with the frame shown in Figure 1(a).

### 2.4. Scanning & Part Positioning

The motorized linear stage (MLS) allows the control of the LTS linear movement along the scan range and the position measurement for each captured image. A uniform mesh of points results from the digitalization. Points belonging to the surface and the contour of significant elements are identified and the measurement tasks are performed. The scan of plane surfaces is allowed by the MLS along its travel range (Table 2).

The work piece is manipulated by a six axis robot. The robot handles the part in order to place the surface being measured in the field of view of the LTS. An initial master piece measurement provides the necessary information to calculate the position change of the robot. It allows the calculation of the position, in the global frame of the part, of the measured surfaces of the production parts without the robot positioning data. In this way, error sources from robot inaccuracy (generally high in robots) are avoided. The dimensions and geometry of the master piece are well-known from its measurement with a CMM. The robot positions in the initial measurement process with the master piece are the same as the robot positions in the measurement of the production parts. The robot brings flexibility to the system due to the high capacity of the robot to position a large number of different parts in the field of view of the LTS.

## 3. Experimental Set Up and Validation

The LTS should be calibrated and the system including the MLS and the LTS must be characterized in order to establish the relationship between the frame of the LTS and the movement direction of the MLS. A high precision gauge object is used to calibrate the LTS and relate it to the MLS.

### 3.1. Characterization Gauge

The characterisation gauge is a high precision object designed and manufactured to allow the calibration of the LTS mounted in the MLS and the validation of the scanning process.

**Figure 5.**
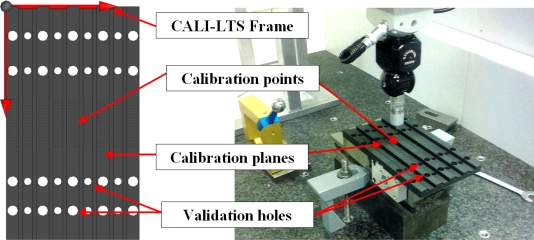
CAD model of the gauge object and measurement in MMC.

The gauge materializes well known nominal coordinates points distributed on different planes for the LTS calibration (Figure 5). The edge of each flat surface allows the characterization of direction of the MLS in the frame of the LTS. The frame of the LTS is defined by the calibration points of the gauge and it is equal to the frame of the gauge in the calibration position (CALI-LTS). The calibration target object allows the system validation by measuring the machined holes on its surface.

A high precision numerically controlled machining centre has been used to machining the part in order to obtain the adequate geometrical precision. In any case, the diameter and position of the holes in the frame of the gauge has been measured with a CMM.

### 3.2. Sensor Modelling and Calibration

**Figure 6.**
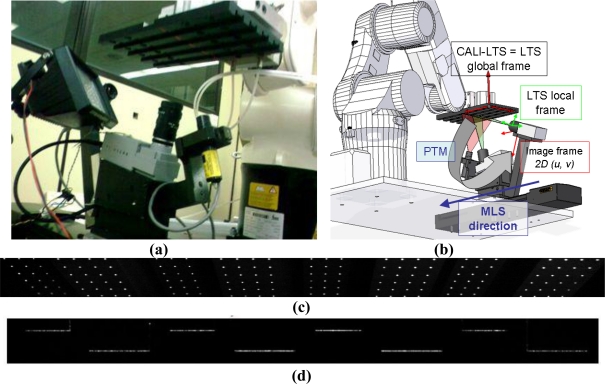
**(a)** Picture of the calibration process. **(b)** Robot in calibration position and reference system in camera calibration. **(c)** Image taken for camera calibration (1280 × 96 px). **(c)** Image taken for laser calibration (1280 × 96 px).

The ideal pin-hole model is used for modelling the cameras. Basically, the camera is modelled with a perspective transformation matrix (PTM). PTM is the change of base matrix (homogeneous matrix sized 4 × 4) needed to transform the known coordinates of a 3D point expressed in the global frame of the LTS into its correspondent 2D coordinates (u, v) in the local frame of the image [Figure (6b)].

Camera and laser models and calibration techniques are well known and widely described in literature, for a detailed description of the PTM construction and LTS calibration see [13,14]. The PTM matrix components consist of the extrinsic parameters related to the CMOS sensor and the lens that define the local frame of the LTS originating in the lens optical centre, and intrinsic parameters that are the component of the transformation matrix relating the global frame of the LTS, defined by the gauge in the calibration, and the local frame of the LTS:

(4) $$\left[\begin{array}{c} s\cdot u\\ s\cdot v\\ s \end{array}\right]=\mathrm{PTM}\cdot \left[\begin{array}{c} X_{\mathrm{LTS}}\\ Y_{\mathrm{LTS}}\\ Z_{\mathrm{LTS}}\\ 1 \end{array}\right];\mathrm{PTM}=\left(\begin{array}{cccc} m_{11} & \cdots & & m_{14}\\ \vdots & \ddots & & \vdots \\ m_{31} & \cdots & & m_{34} \end{array}\right)$$

The equation of a straight line (5) connecting a point whose coordinates are known in the global frame of the LTS (*X_LTS_ Y_LTS_ Z_LTS_*), and its correspondent image point projection with (*u, v*) coordinates in the frame of the image, could be written from (4):

(5) $$\left\{\begin{array}{l} m_{11}\cdot X_{\mathrm{LTS}}+m_{12}\cdot Y_{\mathrm{LTS}}+m_{13}\cdot Z_{\mathrm{LTS}}-m_{31}\cdot u\cdot X_{\mathrm{LTS}}+m_{32}\cdot u\cdot Y_{\mathrm{LTS}}+m_{33}\cdot u\cdot Z_{\mathrm{LTS}}-u\cdot m_{34}+m_{14}=0\\ m_{21}\cdot X_{\mathrm{LTS}}+m_{22}\cdot Y_{\mathrm{LTS}}+m_{23}\cdot Z_{\mathrm{LTS}}-m_{31}\cdot v\cdot X_{\mathrm{LTS}}+m_{32}\cdot v\cdot Y_{\mathrm{LTS}}+m_{33}\cdot v\cdot Z_{\mathrm{LTS}}-v\cdot m_{34}+m_{24}=0 \end{array}\right\}$$

where *m_ij_* is the PTM component of the *i^th^* row and the *j^th^* column.

Since the PTM is a non-invertible matrix, the straight line equation shown in (5) allows calculating, with the laser plane Equation (6) known in the global frame of the LTS, the 3D (*X_LTS_ Y_LTS_ Z_LTS_*) coordinates of a point belonging to the laser line in image with (u,v) coordinates:

(6) $$\mathrm{cA}\;\;X_{\mathrm{LTS}}+\mathrm{cB}\;\;Y_{\mathrm{LTS}}+\mathrm{cC}\;\;Z_{\mathrm{LTS}}+\mathrm{cD}=0;$$

In the calibration process (Figure 6) the global frame of the LTS is defined obtaining the PTM components with the gauge object [calibration points, Figure (6c)], applying linear techniques [33], and the laser plane equation is calculated [calibration planes, Figure (6d)]. After the calibration, a gauge object scanning in the calibration position is used to calculate the MLS movement direction in the global frame of the LTS (Section 3.3).

Once the PTM is known, the coordinates of the calibration points in the image can be recalculated (*u_R_, v_R_*) on the basis of the known coordinates in the global frame of the LTS, as it is indicated in (7):

(7) $$\left(\begin{array}{c} s\cdot u_{R}\\ s\cdot v_{R}\\ s \end{array}\right)=\left(\begin{array}{c} C_{1}\\ C_{2}\\ C_{3} \end{array}\right)=\mathrm{PTM}\cdot \left(\begin{array}{c} X_{\mathrm{LTS}}\\ Y_{\mathrm{LTS}}\\ Z_{\mathrm{LTS}}\\ 1 \end{array}\right)\Rightarrow \{\begin{array}{c} u_{R}=\frac{C_{1}}{C_{3}};\\ v_{R}=\frac{C_{2}}{C_{3}}; \end{array}$$

Therefore, the error obtained in estimating the matrix parameters can be calculated by the difference between the initial point (*u, v*) and the recalculated (*u_R_, v_R_*). In order to verify the performed calibration, the image coordinates corresponding to the calibration points have been recalculated with mean error of 0.68 pixels for coordinate *u* and 0.095 for coordinate *v* for camera 1 and 0.45 pixels for coordinate *u* and 0.51 for coordinate *v* for camera 2.

Once the LTS is calibrated the information provided from the camera (5) for each point of the laser line in image and the equation of the laser plane (6), allows writing a determinate system to solve (*X_LTS_ Y_LTS_ Z_LTS_*) coordinates of the laser line points from its coordinates (*u, v*) in image (8):

(8) $$\left(\begin{array}{ccc} m_{11}-m_{31}\cdot u & m_{12}-m_{32}\cdot u & m_{13}-m_{33}\cdot u\\ m_{21}-m_{31}\cdot u & m_{22}-m_{32}\cdot v & m_{23}-m_{33}\cdot v\\ \mathrm{cA} & \mathrm{cB} & \mathrm{cC} \end{array}\right)\cdot \left(\begin{array}{c} X_{\mathrm{LTS}}\\ Y_{\mathrm{LTS}}\\ Z_{\mathrm{LTS}} \end{array}\right)=\left(\begin{array}{c} u-m_{14}\\ v-m_{24}\\ -\mathrm{cD} \end{array}\right)$$

### 3.3. Motorized Linear Stage Integration

The MLS moves the LTS during the scan. The movement direction of the MLS slightly differs from *Y* direction of the global frame of the LTS and knowing the direction of the MLS in the global frame of the LTS is critical for an accurate reconstruction of the points in image. Several applications use an external measurement device (such as a CMM or a Laser Tracker) to relate the movement direction of the MLS with the global frame of the LTS [13]. In this paper a new integration method to measure de movement direction using only the LTS itself, the MLS and the gauge object, avoiding external measurement devices, is presented. The results are validated and the calculated direction is applied to the measurement of different surfaces of the workpiece in the complete system test.

Laser points in image are reconstructed and a translation *T_i_* = [*x y z*]' is applied to each *i*-th point in order to obtain the surface digitalization. *T_i_* is the displacement of the LTS from the reference position (*P_0_*) to the position of the *i*-th image (9). The calibration position of the LTS is the initial reference position and is therefore a known position:

(9) $${\underline{T}}_{i}=\Delta L_{i}\cdot \left[\begin{array}{l} \text{cos}(\alpha )\\ \text{cos}(\beta )\\ \text{cos}(\gamma ) \end{array}\right];$$

Δ*L_i_* is the difference between the i-th MLS position (*P_i_*) and the reference position data (*P_0_*) (10) and cos(*α*), cos(*β*) and cos(*γ*) are the director cosines of the MLS in the LTS global frame and therefore must satisfy (11):

(10) $$\Delta L_{i}=P_{i}-P_{0};$$

(11) $$\text{cos}{\left(\alpha \right)}^{2}+\text{cos}{\left(\beta \right)}^{2}+\text{cos}{\left(\gamma \right)}^{2}=1;$$

**Figure 7.**
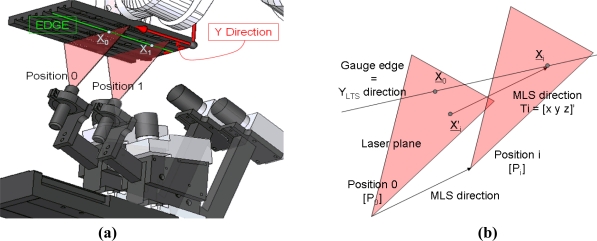
**(a)** Scanning of the gauge for calculating the MLS direction. **(b)** Schematic detail of the *X_i_* projection in the laser plane located at the reference position (*X′_i_*), the direction of the projection is the MLS direction.

The model for calculating the direction of movement is based in the fact that the edges of every flat surface in the gauge materialize the *Y* direction of the frame of the LTS, as the gauge remains in the calibration position, Figure 7. First, the gauge object is scanned in the calibration position. 1300 images are obtained, including the image captured in the reference position and the *i*-th image in the position *P_i_* (Figure 7). One of the corresponding points of one of the edges of the flat surface (u, v coordinates) is located through image analysis in the image captured in the reference position (*P_0_*). The point is reconstructed obtaining *X_0_* = [*x_0_*, *y_0_*, *z_0_*] expressed in the global frame of the LTS.

If the location and reconstruction process of the points in the edges is repeated in each of the 1300 images (image *i* captured in the *i*-th position *P_i_* and *P_i_* is *ΔL* to *P_0_* (10)), *X′_i_* 3D coordinates are obtained [*x′_i_, y′_i_, z′_i_*]. *X′_i_* is the *X_i_* projection on the laser plane in position *P_0_* in the direction of movement of the MLS (12):

(12) $${\underline{X}}_{i}={\underline{X^{\prime }}}_{i}+{\underline{T}}_{i};\{\begin{array}{c} x_{i}={x^{\prime }}_{i}+\Delta L\cdot \text{cos}(\alpha );\\ y_{i}={y^{\prime }}_{i}+\Delta L\cdot \text{cos}(\beta );\\ z_{i}={z^{\prime }}_{i}+\Delta L\cdot \text{cos}(\gamma ); \end{array}$$

As the located points in the 0 image and in the *i*-th image belongs to the same edge, and the edge is aligned with the *Y* direction (frame of the LTS), (13) must be satisfied:

(13) $$x_{0}=x_{i}yz_{0}=z_{i};$$

Finally, taken in consideration (9)–(13), equations system (14) could be written as:

(14) $$\left\{ \begin{array}{l} \frac{{x\prime }_{i}-x_{0}}{\Delta L}=\text{cos}(\alpha );\\ \frac{{z\prime }_{i}-z_{0}}{\Delta L}=\text{cos}(\beta );\\ \text{cos}{\left(\alpha \right)}^{2}+\text{cos}{\left(\beta \right)}^{2}+\text{cos}{\left(\gamma \right)}^{2}=1; \end{array}\right.$$

From (14) the director cosines of the direction of movement of the MLS, expressed in global frame of the LTS, are obtained and the surface reconstruction is enabled.

### 3.4. Validation

The method explained in Section 3.3 is applied to the 1,300 images captured in the scanning of the gauge object. Sixteen edge points are available in each image captured with camera 1 (2 edge points are available in each image captured with camera 2) and (14) is applied for each point located in each edge for all the captured images. A director cosines mean value is calculated using the 1,300 images resulting 16 different values for camera 1 (one value for each edge) and 2 values for camera 2.

**Figure 8.**
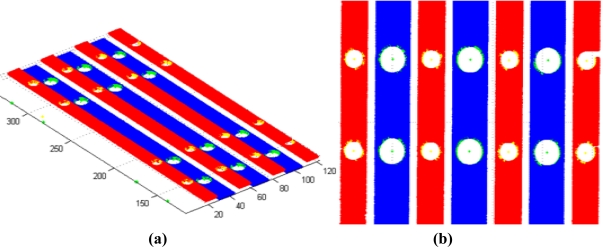
**(a)** Reconstructed cloud points from the gauge digitalization using the selected director cosines. **(b)** Detail of the validation holes in the gauge.

In order to select the direction values with less accumulated error in the process, the distance between the validation holes is measured using each of the MLS directions calculated (Figure 8). In the reconstructed clouds of points the gauge holes are segmented and measured and the measurement results are compared with the measurement using a CMM. The direction selected is the one that minimizes the distance error between the centres measured using a CMM and the ones reconstructed with the LTS, Table 4.

To obtain the MLS direction with the camera 2, the same process as with camera 1 is followed using the camera 2 images.

## 4. Operation Process

**Figure 9.**
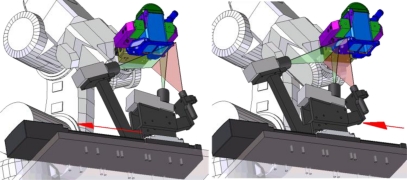
Scanning process.

**Figure 10.**
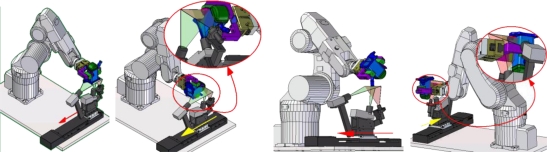
Positioning of the workpiece to measure each element.

Once calibration of the LTS has been performed with the captured images of the LTS-gauge, the points in the laser line are known in the global frame of the LTS and the part surfaces can be scanned (Figure 9) using a robot to positioning each surface in the field of view of the LTS (Figure 10). This section shows a method for measuring the different surfaces of the part and establishes the position of each element in the frame of the part avoiding using robot data.

The measurement process is divided in six stages: data acquisition, image analysis, reconstruction of the cloud points and analysis for each element of the part, coordinate system transformation to express point coordinates of each element in the reference system of the part and, finally, result analysis. This section is focused in the reconstruction of the cloud points and the coordinate system transformation to express point coordinates of each element into the global frame of the part.

### 4.1. Surfaces Reconstruction

As it is mentioned in Section 3, the displacement and the direction of the MLS has to be taken into account to an appropriate surface reconstruction from the laser line points of each image. For each point in image identified as a surface point, the coordinates in the frame of the LTS have to be calculated and after that, the translation T has to be applied as shown in (15):

(15) $${\underline{X}}_{i,j}={\underline{X}}_{i,j}\prime +{\underline{T}}_{j};$$

where:

- *X_i,j_*: *i*-th point of the *j*-th image;
- *X_i,j_′: i*-th point of the *j*-th image projection under the MLS direction in the laser plane in position P_0_.
- *T_j_*: defined in (12), MLS translation between the *j*-th position and the reference position P0 expressed in the frame of the LTS.

**Figure 11.**
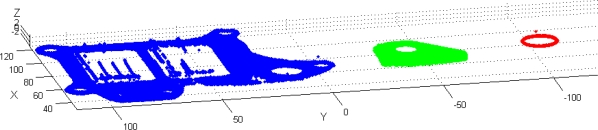
Different part surfaces reconstructed before the frame transformation to express each point coordinates into the global frame of the part (camera 1).

Before applying the model for the integration of the robot the cloud points of each scanned element appears along the LTS-MLS travel range in the scanning position (Figure 11).

### 4.2. Master Piece & Robot Integration

**Figure 12.**
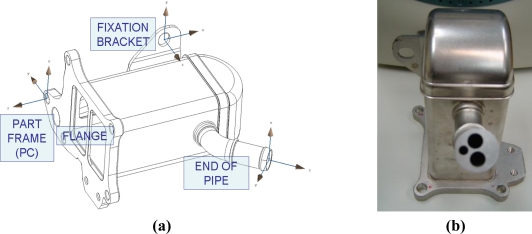
**(a)** Elements local frames in the master piece. **(b)** Master piece end of pipe.

In order to reduce the effect of robot errors the implemented method considers the use of a master piece, measured with a CMM, to obtain the transformation matrices of each local coordinate system, of each surface to be measured, to the global frame of the part (Figure 12). The initial measurement of a master piece avoids the use of the robot data to link the scanning point coordinates of the *n*-th element with the global frame of the part.

**Figure 13.**
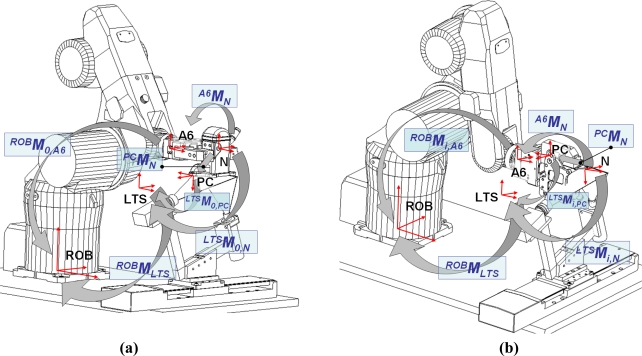
Principal frames and transformation matrices used in the proposed model. The same pattern is applied to the serial piece substituting PC with pc & N with n. **(a)** Measurement of the element that materializes the global frame of the part, robot position 0. **(b)** Measurement of the *n*-th element, robot position *i*.

The transformation matrix between the *n*-th element and the frame of the part could be written as (16) using the transformation matrix that links the frames in Figure 13(a), where the system is scanning the global frame of the part (robot position 0):

(16) $${}^{\mathrm{pc}}M_{n}={}^{\mathrm{LTS}}M_{0,\mathrm{pc}}^{-1}\cdot {}^{\mathrm{ROB}}M_{\mathrm{LTS}}^{-1}\cdot {}^{\mathrm{ROB}}M_{0,A6}\cdot {}^{A6}M_{n}$$

Equation (16) is a product of matrices where *^FrameB^M_RobotPosition,FrameA_*, is a 4 × 4 homogeneous change of base matrix to transform the points coordinates known in the frame A into the frame B; the robot position indicates if the measure element is the flange (position 0, e.g., *^ROB^M_0,A6_*), where the global frame of the part is defined, or other element (position *i* with *i* = 1 to the number of elements to link with the global frame of the part); when no robot position is referred the values of *M* do not depend of the position of the robot end effector (e.g., *^ROB^M_LTS_*). The frames involved in the developed method are shown in Table 5.

In (16) *^LTS^M_0,pc_* is calculated from the scanned cloud points but the other matrices are unknown. It is possible to write the same links for the measurement of another element with the robot in position *i* (17), Figure 13(b):

(17) $${}^{\mathrm{pc}}M_{n}={}^{\mathrm{LTS}}M_{i,\mathrm{pc}}^{-1}\cdot {}^{\mathrm{ROB}}M_{\mathrm{LTS}}^{-1}\cdot {}^{\mathrm{ROB}}M_{i,A6}\cdot {}^{A6}M_{n}$$

If the measured part is the master piece (16) and (17) could be written as (18) and (19):

(18) $${}^{\mathrm{PC}}M_{N}={}^{\mathrm{LTS}}M_{0,\mathrm{PC}}^{-1}\cdot {}^{\mathrm{ROB}}M_{\mathrm{LTS}}^{-1}\cdot {}^{\mathrm{ROB}}M_{0,A6}\cdot {}^{A6}M_{N};\;\text{Robot position}\;0$$

(19) $${}^{\mathrm{PC}}M_{N}={}^{\mathrm{LTS}}M_{i,\mathrm{PC}}^{-1}\cdot {}^{\mathrm{ROB}}M_{\mathrm{LTS}}^{-1}\cdot {}^{\mathrm{ROB}}M_{i,A6}\cdot {}^{A6}M_{N};\;\text{Robot position}\;i$$

where the capital letters indicate that the elements (PC or N) belong to the master piece.

Since the master piece is measured with a CMM, the transformation matrix between each element and the global frame of the part is known, *^PC^M_N_*. As it appears in Figure 12(b), a circular gauge with three holes is inserted in the pipe of the master piece to materialize a measurable local frame. *^LTS^M_0,PC_* is calculated from the scanned cloud points, as *^LTS^M_0,pc_* in (16) and *^LTS^M_i,PC_* can be calculated (20):

(20) $${}^{\mathrm{LTS}}M_{i,\mathrm{PC}}^{-1}={}^{\mathrm{PC}}M_{N}\cdot {}^{\mathrm{LTS}}M_{i,N}^{-1}$$

*^LTS^M_i,N_* is obtained from the scanned cloud points.

Grouping (18)–(20) the transformation matrices referred to the frames of the robot can be expressed as a known matrices product (21):

(21) $${}^{\mathrm{ROB}}M_{\mathrm{LTS}}^{-1}\cdot {}^{\mathrm{ROB}}M_{0,A6}\cdot {}^{\mathrm{ROB}}M_{i,A6}^{-1}\cdot {}^{\mathrm{ROB}}M_{\mathrm{LTS}}={}^{\mathrm{LTS}}M_{0,\mathrm{PC}}\cdot {}^{\mathrm{PC}}M_{N}\cdot {}^{\mathrm{LTS}}M_{i,N}^{-1}$$

The matrices in the right side of (21) are known and referred to the master piece measurement.

The same equation development could be written for the remaining parts measurement (serial pieces) (22):

(22) $${}^{\mathrm{ROB}}M_{\mathrm{LTS}}^{-1}\cdot {}^{\mathrm{ROB}}M_{0,A6}\cdot {}^{\mathrm{ROB}}M_{i,A6}^{-1}\cdot {}^{\mathrm{ROB}}M_{\mathrm{LTS}}={}^{\mathrm{LTS}}M_{0,\mathrm{pc}}\cdot {}^{\mathrm{pc}}M_{n}\cdot {}^{\mathrm{LTS}}M_{i,n}^{-1}$$

The left side of (22) is equal to the left side of (21) because the robot positions to measure each element of the master piece are the same that the ones used to measure the serial part, so *^pc^M_n_* can be calculated avoiding the robot data utilization (23):

(23) $${}^{\mathrm{pc}}M_{n}={}^{\mathrm{LTS}}M_{0,\mathrm{pc}}^{-1}\cdot {}^{\mathrm{LTS}}M_{0,\mathrm{PC}}\cdot {}^{\mathrm{PC}}M_{N}\cdot {}^{\mathrm{LTS}}M_{i,N}^{-1}\cdot {}^{\mathrm{LTS}}M_{i,n}$$

The precision of the method depends on the robot repeatability because the same reached position is considered for the master piece and the rest of parts (serial pieces), so a repeatability test has been performed with the robot in order to evaluate the lack of repeatability effect in the measurement results.

### 4.3. Robot Repeatability Test

**Figure 14.**
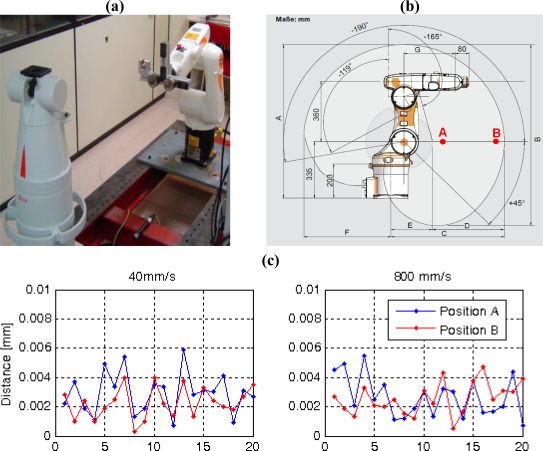
Repeatability test. **(a)** Laser tracker and robot during the test. **(b)** Schematic situation of points A and B. **(c)** Repeatability test results: distance between the position reached in each iteration and the mean position calculated with the twenty iterations.

A repeatability test [34] has been performed at different speeds to reach points A and B shown in Figure 14. The distance between A and B was 300 mm and 20 iterations where made positioning the robot at each point. A laser tracker was used to measure the reached positions. For the speed tested the positioning repeatability results remain under 6 μm. Trials were carried out with the tracker retroreflector in the center and the periphery of the support of the robot, giving repeatability of the order of the previous ones and therefore lower orientation errors.

The repeatability value obtained from the test, indicate an acceptable effect of the robot positioning repeatability in the measurement results.

## 5. Test and Results

Precision has been studied using a reference part. This was a heat exchanger with several elements to be verified, set in various positions and orientations, as shown in Figure (12a). The conditions in which the test took place were similar to those found when measuring parts in industrial facilities. The image and 3D cloud points processing software have been developed in order to work correctly with a variety of components other than the reference part. However, it should be pointed out that the system behavior is highly sensitive to the features of the measured surfaces, their reflectivity, and the contour of the location elements (round or countersunk holes), and that variations in such characteristics have been taken into account.

The flatness of the flange and the fixation bracket are checked with the camera 1. With the camera 2, the position of the mounting hole of the fixation bracket and the end of the pipe is verified. The dimensions of the flange are 125 × 96 mm of flat surface with holes and windows for the circulation of fluids and fastening the exchanger. The size of the fixation bracket is 45 × 28 mm, and it has an 11 mm-long and 5 mm radius mounting hole at its center. Thus, position of the center of this mounting hole is measured. And finally, the diameter of the end of the tube is 16 mm.

**Figure 15.**
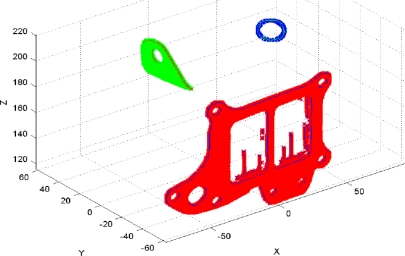
Several iterations reconstructed.

The system accuracy has been studied by measuring the reference part ten times and analyzing the variation in the results regarding their mean value. The digitalization results for several such iterations are shown superimposed on Figure 15. Accuracy was studied by comparing the results of the measurement system with the results when measuring the same characteristics of the part using a CMM.

In order to evaluate the system measuring diameter of holes, position of components, and so on, different representative parameters are selected. Camera 1 checks the flatness of the surfaces and the representative parameter, flatness in this case, will be used to evaluate the accuracy and repeatability of the system. The theoretical plane is calculated as that fitted by the object points (flange or fixation bracket) by means of least squares. As well as precision, it is possible to perform qualitative analysis of the flatness inspection, based on the representation of the distance from the theoretical plane of each object points using a color scale.

**Figure 16.**
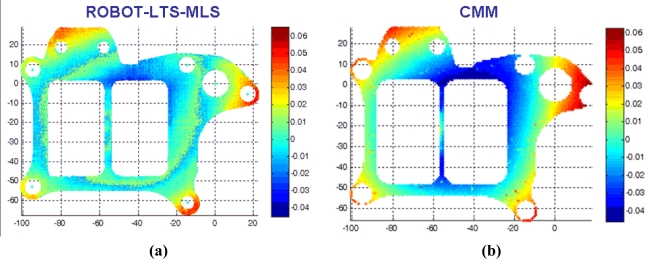
Distance from the theoretical plane of the points of the measured flange. **(a)** Points digitalized with the Robot-LTS system. **(b)** Contact probed points in the CMM.

Once the iterations have been carried out, and after analyzing the measurement images and the digitalized points, the flatness results shown in Figure 16 are obtained for the flange. As mentioned above, the distance from the theoretical plane of each point on the flange is represented by a color scale. This distance is calculated using the Robot-MLS-LTS integrated system, and next it can be appreciated the distance between each point and the plane as measured using a CMM and the same color scale.

**Figure 17.**
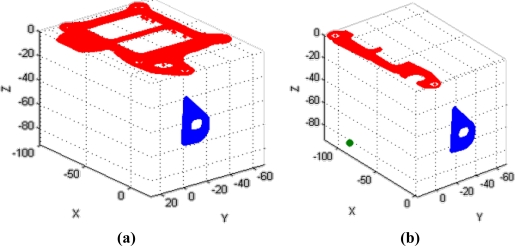
Reconstruction of the scanned cloud points in the global frame of the part. **(a)** Camera 1 scanning. **(b)** Camera 2 scanning.

Similar results are obtained for the fixation bracket. The coordinates of the reconstructed points of each element expressed in the global frame of the part are graphed in Figure 17.

Analyzing the results obtained by measuring ten times the reference part, the following indicators for the characteristics uncertainties for different kind of measures can be concluded:

- Flatness: 0.020 mm
- Position/diameter in the same item: 0.030 mm
- Position between other items: 0.060 mm

These values have been calculated according with the GUM [35] using a confidence level of 95% (k = 2). Finally, the process takes 20 s for the data acquisition and 4 s for the complete data analysis of the final results.

## 6. Conclusions

This article presents the design analysis, model and test of a novel sensor based in laser triangulation using two cameras. The scanning process is carried out by a motorized linear stage on which the sensor is mounted. Using a robot to positioning different part surfaces within the field of view of the sensor, allows the surfaces measurement expressing the results in the global frame of the part. A method for the calculation of the direction of movement of the motorized linear stage in the global frame of the part has been developed and validated with a gauge object. The method avoids the direct direction measurement by external devices simplifying the measurement system set up and allowing the self recalibration system on the inspection line.

The measurement model developed takes the robot as a positioning element and uses a master piece of known dimensions to set the relative positions of the robot in the measurement of each surface. This model takes advantage of the robot flexibility avoiding its inaccuracy. Test results confirm the accuracy and repeatability of the complete system for measuring different components and characteristics of a reference model, with appropriate repeatability values for checking complex geometry parts and adequate cycle time to allow the 100% production inspection.

## Figures and Tables

**Table 1.** Specifications of the LTS.

**Characteristic**	**Size**	**Tolerance**
Surface Flatness	110 × 200 mm^2^	0.15 mm
Diameter of holes in the surface	6–20 mm	0.2 mm

**Table 2.** Components list.

Component	Pcs.	Characteristics
Camera	2	CMOS sensor 1024 × 1280 px. Selectable Region Of Interest, ROI (96 px in v coordinate). Frame rate106 fps at selected ROI.
Lens	1	High resolution lens for 2/3″ sensors, focal distance *f* = 12 mm, minimum object distance MOD = 150 mm & F1.4-close. θ_h_ = 38°3′, θ_v_ = 26°2′.
	1	High resolution lens for 2/3″ sensors, focal distance *f* = 35 mm, MOD = 200 mm & F12.0-16. θ_h_ = 14°4′, θ_v_ = 10°8′.
Optic Filter	2	Interferential filter λ_c_ = 660 nm, bandwidth 20 ± 2 nm.
Laser	1	Laser diode generator, λ = 660 nm, 5mW, Class II > 1 mW.
Optic Pattern	1	Laser line generator with uniform (non-Gaussian) lengthwise.
Motion Linear Stage	1	DC servo motor, travel range 250 mm, maximum speed 50 mm/s, 4,000 pts/rev. encoder located directly on the screw resolution 0.5 μm, accuracy 5 μm (typical 2.5 μm), uni-directional repeatability 1.5 μm.
Robot manipulator	1	Six axis anthropomorphic robot, reach of 650 mm, payload of 5 kg. Repeatability < ±0.02 mm according with ISO 9283. Maximum speed 8.2 m/s.

**Table 3.** Resolution of the images [mm/pixel]. *X*, *Y* & *Z* directions are shown in Figure 1a.

**Device**	**X resolution [mm/px]**	**Y resolution [mm/px]**	**Z resolution [mm/px]**
Camera 1 Image	0.10	0.05	0.08
Camera 2 Image	0.02	0.04	0.11

**Table 4.** MLS direction cosines obtained for each camera.

	**cos(*α*)**	**cos(*β*)**	**cos(*γ*)**
Camera 1	−0.003	0.999	−0.002
Camera 2	−0.004	0.999	−0.002

**Table 5.** Principal frames used in the proposed method.

**Name**	**Frame**
ROB	Global frame of the robot.
A6	Local frame of the robot end effector.
PC* or pc	Global frame of the Part.
N* or n	Local frame of a part element.
LTS	Global frame of the LTS.

* In capital letter refers to the master piece.
